# Mapping of quantitative trait loci underlying a magic trait in ongoing ecological speciation

**DOI:** 10.1186/s12864-021-07908-4

**Published:** 2021-08-12

**Authors:** Tetsumi Takahashi, Atsushi J. Nagano, Teiji Sota

**Affiliations:** 1 Institute of Natural and Environmental Sciences, University of Hyogo, Sanda, Hyogo 669-1546 Japan; 2 Division of Nature and Environmental Management, Museum of Nature and Human Activities, Sanda, Hyogo 669-1546 Japan; 3 Faculty of Agriculture, Ryukoku University, Otsu, Shiga 520-2194 Japan; 4 Graduate School of Science, Kyoto University, Sakyo, Kyoto, 606-8502, Japan

**Keywords:** Body size, Cichlid fish, Lake Tanganyika, Polygenic inheritance, *Telmatochromis temporalis*

## Abstract

**Background:**

*Telmatochromis temporalis* is a cichlid fish endemic to Lake Tanganyika. The normal and dwarf morphs of this fish are a clear example of ongoing ecological speciation, and body size plays an important role in this speciation event as a magic trait. However, the genetic basis underlying this trait has not been studied.

**Results:**

Based on double-digested restriction-site associated DNA (ddRAD) sequencing of a hybrid cross between the morphs that includes F0 male, F0 female, and 206 F2 individuals, we obtained a linkage map consisting of 708 ddRAD markers in 22 linkage groups, which corresponded to the previously reported *Oreochromis niloticus* chromosomes, and identified one significant and five suggestive quantitative trait loci (QTL) for body size. From the body-size distribution pattern, the significant and three of the five suggestive QTL are possibly associated with genes responsible for the difference in body size between the morphs.

**Conclusions:**

The QTL analysis presented here suggests that multiple genes, rather than a single gene, control morph-specific body size. The present results provide further insights about the genes underlying the morph specific body size and evolution of the magic trait during ecological speciation.

**Supplementary Information:**

The online version contains supplementary material available at 10.1186/s12864-021-07908-4.

## Background

Ecological speciation is the process by which barriers to gene flow evolve between populations as a result of ecological based divergent selection between environments [1, 2]. The most direct way to link divergent natural selection to reproductive isolation is via ‘magic traits’, i.e., traits under selection that also contribute to non-random mating or genes under selection that pleiotropically affect non-random mating [2, 3]. In many species, body size is a life-history trait that has a serious impact on individuals’ fitness through natural or sexual selection [4–6]. This suggests that body size could be a candidate magic trait. In some species, body size is thought to be a ‘classic’ magic trait, in that body size evolved under divergent selection and also acted on mating cues, although there are few conclusive examples [3]. Body size may also act as an ‘automatic’ magic trait, where under selection it automatically leads to assortative mating via, e.g., geographical segregation [3]. A clear example of such size-mediated automatic magic traits is reported for *Telmatochromis temporalis* from Lake Tanganyika.

Lake Tanganyika is the oldest lake in the African Great Rift Valley. Over thousands of years, lakes generally become filled with lacustrine deposits. However, Lake Tanganyika deepens faster than sedimentation occurs due to underlying plate tectonics and has been filled with water for the past 9–12 million years [7]; thus, this lake is classified as an ancient lake [8]. Lake Tanganyika harbours approximately 250 cichlid species, most of which are endemic to the lake and are morphologically, ecologically, and genetically diverse [9, 10]. These fish are derived from a single ancestral species and are thought to have evolved through explosive adaptive radiation in the lake or pre-existed water systems [11, 12].

**Fig. 1:**
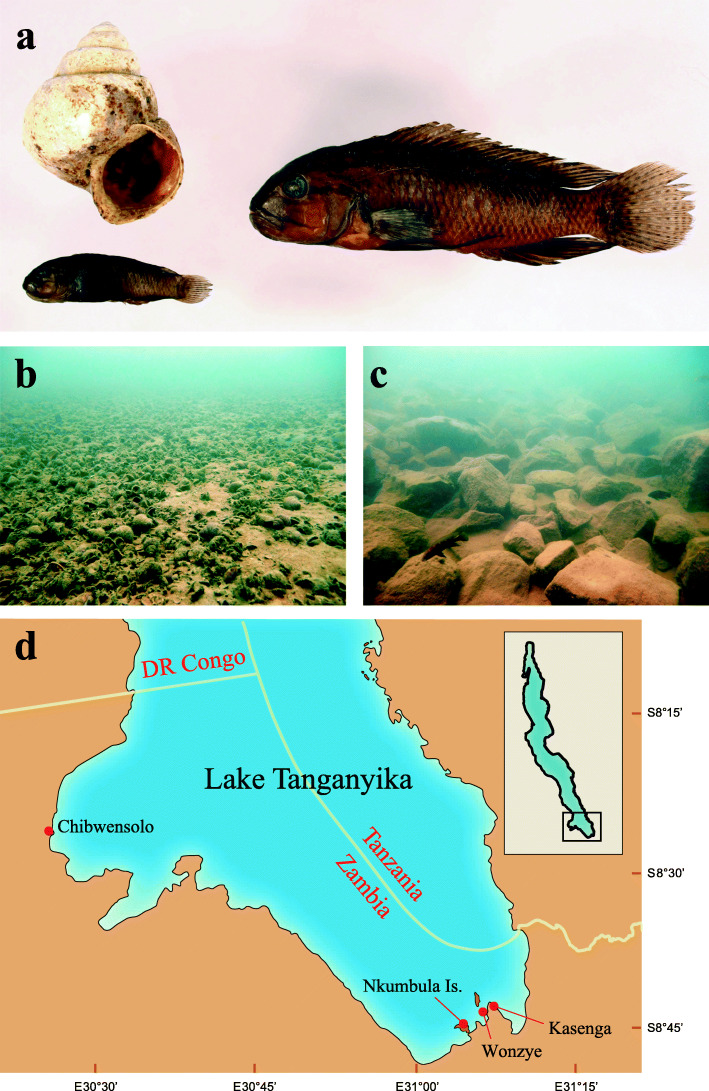
*Telmatochromis temporalis* and their habitats. **a** Mature males of the dwarf (left) and normal (right) morphs collected at Wonzye, Zambia. The dwarf male used the snail shell as shelters. **b** Shell bed at Wonzye. **c** Rocky habitat at Wonzye. **d** A map of the southern end of Lake Tanganyika

*Telmatochromis temporalis* is an algae-feeding cichlid fish endemic to Lake Tanganyika. This fish consists of two main morphs (Fig. 1a) [13]. The normal morph has a large body size [the body size differs somewhat among populations, and in Wonzye, Zambia (Fig. 1d), ranges between 56.4 and 75.7 mm in standard length (SL) in adult males and 33.4–53.1 mm SL in adult females] [14]. This morph inhabits rocky bottoms (Fig. 1c) and usually uses spaces under the rocks to hide from predators and spawn. The dwarf morph is smaller (in Wonzye, the body size ranges between 25.1 and 40.3 mm SL in adult males and 17.2–26.8 mm SL in adult females) [14]. This morph uses empty snail shells of *Neothauma tanganyicense* to hide and spawn in shell beds, where a lot of empty snail shells exist on sandy bottoms (Fig. 1b). Although the normal morph and rocky bottoms are widely distributed in the shallow waters along the lake shores, the dwarf morph and shell-beds are restricted to the Wonzye–Nkumbula area and around Chibwensolo in Zambian waters; these areas are approximately 80 km apart in a straight line (Fig. 1d). The normal and dwarf morphs are genetically close within the same area but distant between areas [15, 16]. A population genetics study suggested that the dwarf morph had evolved independently from the normal morph in these areas [17]. Another morph, called as ‘slender’, was recently reported from Kasenga, Zambia [18]. This morph is genetically close to the normal morph from the same locality, and no evidence suggests that this morph has affected the evolution of the dwarf morph.

The difference in body size between the normal and dwarf morphs might have evolved through divergent natural selection because the body sizes correlate closely with the sizes of available hiding spaces in their habitats [15]. Details of evolution of the derived small body size have been studied in the dwarf morph from Wonzye [14, 19]. Larger males control more females in their territories, suggesting a strong sexual selection for body size. However, having an extremely large body size is disadvantageous for the fish. They enter the shells head-first to hide from predators. Large males ( > ~ 35 mm SL) cannot turn within the shells and must exit tail-first, making it difficult for them to visually confirm that it is safe to venture outside (i.e. the presence of predators). The males’ body size appears to have evolved to balance between these sexual and natural selection pressures. In dwarf females, the evolution of body size may be due to fecundity and natural selection; larger females can spawn more eggs, but smaller females can deposit their eggs in narrower, safer spaces within shells.

Gene flow is restricted between normal and dwarf morphs from the same locality, even if no obvious geographical barriers exist between the habitats [15, 16]. This reproductive isolation may be due to the body-size difference. A habitat-choice experiment in tanks showed that large individuals ( > ~ 40 mm SL) tended to prefer rocky habitats, whereas small individuals ( < ~ 40 mm SL) tended to prefer shell-bed habitats [16]. This habitat preference may have resulted in the discrete distribution observed in the wild, with the normal morph adults in rocky habitats and dwarf morph adults in shell-bed habitats.

Overall, the body size of *T. temporalis* has most likely evolved under divergent natural selection [15] and led to reproductive isolation between normal and dwarf morphs via geographic segregation [16]. Therefore, body size can be regarded as an automatic magic trait during ecological speciation. A common garden experiment indicated that the difference in body size between the morphs was not exclusively a consequence of phenotypic plasticity [16]. However, the genetic basis of the difference in body size has not been investigated. In this study, we conducted a double-digested restriction-site associated DNA (ddRAD) sequencing of a hybrid cross between the normal and dwarf morphs to construct a linkage map and identify quantitative trait loci (QTL) underlying body size variation.

## Results

### Processing of ddRAD sequences

One lane of paired-end sequencing on an Illumina HiSeq X produced a total of 401 million ddRAD tag sequence pairs from the normal F0 male, dwarf F0 female, and 206 F2 individuals. The number of ddRAD sequence pairs per sample ranged from 2.7 × 10^5^ to 7.0 × 10^6^ (1.9 × 10^6^ on average) [DNA Data Bank of Japan (DDBJ) accession no. DRA011699]. The mean merged coverage depth in the *de novo* assembly was 74.2 for the F0 male and 82.4 for the F0 female and ranged from 18.5 to 95.2 (56.0 on average) for the F2 individuals. A total of 55,209 orthologous loci were obtained from an orthologous search between the F0 male and F0 female, of which 14,528 were polymorphic. The F2 individuals were genotyped for the polymorphic loci, and 7,275 loci were recovered from > 60 % of the samples. We identified 1,409 single nucleotide polymorphisms (SNPs) that did not share alleles between the parents. After discarding SNPs with genotypes that extremely departed from the expected segregation pattern, the remaining 709 SNPs were included in the following analyses.

### Linkage map construction

Twenty-two linkage groups (LGs) were obtained from 708 SNPs (one SNP was not assigned to any LGs), which spanned over 1,252 centimorgan (cM) (Supplementary Fig. S1). This number of LGs matches the available data for most African cichlids, although four species of the tribe Lamprologini, to which *T. temporalis* belongs, had 2n = 42 chromosomes [20]. Each LG consisted of 19–66 SNPs (32.2 SNPs on average). The blast search of ddRAD loci containing these SNPs showed that the 22 LGs corresponded to the previously reported LG1–LG23 (no LG21) of Nile tilapia *Oreochromis niloticus* [21] (Supplementary Table S1), which belongs to the subfamily Pseudocrenilabrinae along with *T. temporalis* [22]. The present LGs were therefore numbered in accordance with the *O. niloticus* LGs.

### QTL mapping

**Fig. 2:**
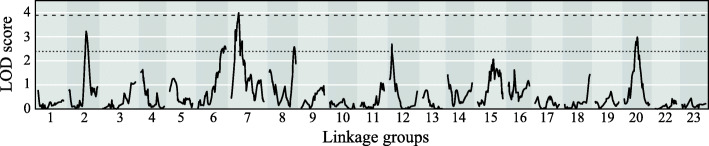
Quantitative trait locus (QTL) plots for body size in *Telmatochromis temporalis*. Broken and dotted lines in the graph indicate significant (3.89) and suggestive (2.39) thresholds, respectively, of the logarithm of odds (LOD) score

**Fig. 3:**
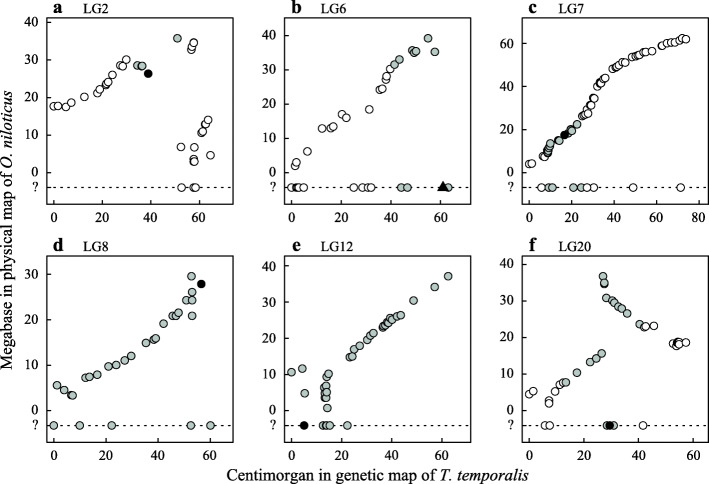
Comparison of double-digested restriction-site associated DNA (ddRAD) locus positions between *Telmatochromis temporalis* and *Oreochromis niloticus*. **a** Linkage group (LG) 2. **b** LG6. **c** LG7. **d** LG8. **e** LG12. **f** LG20. Closed circle: quantitative trait locus (QTL) on a ddRAD locus. Closed triangle: QTL on a pseudomarker. Grey circles: ddRAD loci within the 95 % credible interval. Open circles: the other ddRAD loci. Question mark indicates markers for which the positions on the *O. niloticus* LG were not identified. See Supplementary Fig. S2 for the other LGs

**Fig. 4:**
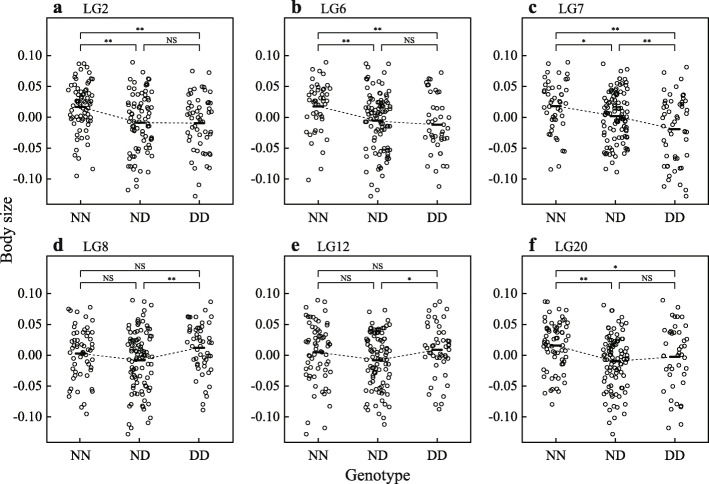
Effect plots for body size at quantitative trait loci (QTL) in *Telmatochromis temporalis*. Body size was expressed by residuals from two-way ANOVA on log_10_(SL). **a** Linkage group (LG) 2. **b** LG6. **c** LG7. **d** LG8. **e** LG12. **f** LG20. NN: homozygotes of normal alleles. ND: heterozygotes. DD: homozygotes of dwarf alleles. Differences in body size between genotypes were tested using t-tests. ** *p* ≤ 0.01, * *p* ≤ 0.05, and NS *p* > 0.05

A QTL analysis for body size identified one peak that exceeded the significant logarithm of odds (LOD) threshold of 3.89 and five peaks that exceeded the suggestive threshold of 2.39 (Fig. 2; Table 1). The QTL peak on LG6 was a pseudomarker. The corresponding position of the pseudomarker on the *O. niloticus* LG6 could not be identified because closely related ddRAD markers were not mapped onto the genome (Fig. 3b). The ddRAD markers at the QTL peaks on LG12 and LG20 were not mapped onto the *O. niloticus* genome (Fig. 3e, f). In LG2 and LG20, the *O. niloticus* genome region that corresponds to the 95 % Bayes credible interval of the QTL was separated into two parts, due to a complex relationship of markers between these species (Fig. 3a, f). The plot of body size indicated the dominance of the QTL on LG2, LG6, and LG20, in which F2 fish with at least one dwarf allele were small (Fig. 4a, b, f), the incomplete dominance of the QTL on LG7, in which F2 individuals homozygous for the normal alleles were large and individuals homozygous for the dwarf alleles were small (Fig. 4c), and intermediate pattern between overdominance and dominance of the QTL on LG8 and LG12, in which F2 individuals homozygous for the dwarf alleles were large and heterozygotes were small (Fig. 4d, e).

**Table 1:** Properties of QTL for *Telmatochromis temporalis* body size and corresponding positions on *Oreochromis niloticus* genome

	*T. temporalis*					*O. niloticus*	
LG	Pos (cM)	Marker type	LOD score	PVE	CI (cM)	Pos (Mb)	CI (Mb)
2	38.9	ddRAD marker	3.22 ^sug^	0.074	34.4–50.9	26.4	26.4–28.5, 35.8
6	61.0	Pseudomarker	2.61 ^sug^	0.057	41.5–63.1	?	31.5–39.2
7	16.7	ddRAD marker	3.99 ^sig^	0.084	8.5–24.5	17.5	9.1–25.5
8	56.5	ddRAD marker	2.57 ^sug^	0.035	0.0–60.0	27.9	3.4–29.6
12	5.0	ddRAD marker	2.69 ^sug^	0.026	0.0–62.5	?	0.7–37.1
20	29.5	ddRAD marker	2.98 ^sug^	0.061	17.5–40.4	?	10.4–15.7, 23.6–36.8

*CI* 95 % Bayes credible interval, *cM* Centimorgan, *ddRAD* Double-digested restriction-site associated DNA, *LG* Linkage group, *LOD* Logarithm of odds, *Mb* Megabase, *Pos* Position of the QTL, *PVE* Proportion of phenotypic variance explained by the QTL, *QTL* Quantitative trait loci, *sig* Significant LOD score > 3.89, *sug* Suggestive LOD score > 2.39

## Discussion

The normal and dwarf morphs of *T. temporalis* are a clear example of ongoing ecological speciation [15, 16]. Body size plays an important role during the speciation between the morphs as a magic trait. The present study identified one significant and five suggestive QTL for this trait, using ddRAD markers generated from a cross between the morphs (Fig. 2; Table 1). Regarding the significant and three of the five suggestive QTL on LG2, LG6, LG7, and LG20, alleles from the dwarf F0 female expressed a smaller body size than did alleles from the normal F0 male (Fig. 4a–c, f). This tendency supports the hypothesis that these QTL are linked to genes responsible for the body-size difference between the morphs. The other suggestive QTL on LG8 and LG12 were associated with an unexpected body-size pattern (Fig. 4d, e); F2 individuals homozygous for the dwarf alleles were larger than the others. As suggestive linkage results can often be wrong [23], these QTL might have been erroneously detected by chance or may be indicative of other type of body-size variance, e.g., variance within a morph. It is worth noting that the QTL for growth traits partly differ between sexes in *Oreochromis* species [24, 25]. This may also be true in *T. temporalis*. However, the present study pooled male and female samples in the analysis to increase the QTL detection power. Actually, when males and females were analysed separately, the results were not clear, probably due to the decreased sample sizes (not shown). Therefore, the present study might have overlooked some QTL skew between the sexes.

An annotated genome assembly for *O. niloticus* is useful for suggesting candidate genes for the body-size difference between the *T. temporalis* morphs. Although their functions in cichlid fishes are not known, some genes responsible for body size in vertebrates were found in (or very close to) the *O. niloticus* genomic regions that correspond to the 95 % credible intervals of the *T. temporalis* QTL, such as early growth response 1 (*egr1*) on LG2 at 26.3 Mb [26, 27], insulin-like growth factor binding protein 4 (*igfbp4*) on LG6 at 39.0 Mb [28, 29], SMAD family member 7 (*smad7*) on LG7 at 18.3 Mb [30], and nuclear receptor subfamily 2 group C member 2 (*nr2c2*) on LG20 at 14.6 Mb [31]. Further studies are needed to confirm the functions of theses genes in *T. temporalis*. Insulin-like growth factor 1 (*igf1*) is a well-known gene responsible for body size in mammals and fish [28, 32–35]. The growth hormone receptor (*ghr*) is reported to influence the expression level of *igf1* and eventually, body size in *Oreochromis* fishes [24]. However, these genes are not located in the *O. niloticus* genome regions corresponding to the credible intervals of the *T. temporalis* QTL, i.e., *igf1* on LG17 at 16.8 Mb and *ghr* on LG7 at 31.9 Mb. Previous QTL mapping and genome-wide association study of *Oreochromis* species reported several genome loci associated with growth traits [24, 25, 36], and some of these loci are within the *O. niloticus* genome regions corresponding to the *T. temporalis* QTL credible intervals, i.e., LG2 at 26.5 Mb for male body thickness [25], LG7 at 16.8 Mb for fillet yield [36], and LG20 at 11.8 Mb for body weight and total length [25]. Some common genes may control body size in these phylogenetically distant species.

We identified four QTL that are supposedly linked to genes responsible for the body-size difference between the normal and dwarf morphs. This suggests that multiple genes, rather than a single gene, control the morph-specific body size. Interestingly, such a polygenic model can explain the mechanism of evolution of the dwarf morph. The dwarf morph, which inhabits patchily distributed shell beds, presumably evolved repeatedly from the normal morph, which is common in shallow waters along the lake coast [17]. As suggested in the present study, individuals with dwarf alleles of a body-size regulating gene are not necessarily small (Fig. 4). Therefore, dwarf alleles can be maintained in the normal morph populations at low frequencies as standing genetic variations. When shell beds emerged next to a population due to water-level changes [37], small body size would be evolved by fixation of the dwarf alleles in these body-size regulating genes under natural selection [16], whereby small individuals would have an advantage of using empty snail shells for sheltering and spawning [14, 19]. This mechanism might have caused the parallel evolution of the dwarf morph at distant shell beds [16, 17]. To confirm this hypothesis, the dwarf alleles of genes responsible for body-size regulation must be identified, and the distributions of dwarf alleles in wild populations of the normal and dwarf morphs should be examined.

## Conclusions

Lake Tanganyika harbours ~ 250 cichlid species, and the diversity of these fishes has been studied in terms of, e.g., morphology, behaviour, ecology, and genetics [10, 38–40]. However, the genetic basis of body size has somewhat been overlooked, despite there being significant body size diversity among and within species [41], e.g., from ~ 4 cm SL in *Neolamprologus multifasciatus* to ~ 50 cm SL in *Boulengerochromis microlepis*. The only existing study found that a single-locus two-allele polymorphism in a sex-linked chromosome in heterogametic males controlled body-size difference between giant bourgeois males and miniature males in *Lamprologus callipterus*, which display different reproductive tactics [42]. The present study identified four genomic loci that are possibly associated with the body-size difference between the normal and dwarf morphs of *T. temporalis*. Identification of genes regulating body size in this fish species will enable further understanding of the mechanisms underlying size-mediated ecological speciation and provide insight into the recent explosive adaptive radiation that had occurred in this lake.

## Materials and methods

### Fish cross for QTL analysis

A normal F0 male and a dwarf F0 female of *T. temporalis* from Wonzye, Zambia were crossed in a tank to produce the F1 generation. Two F1 tanks, each containing one F1 male and two F1 females, were set up to produce the F2 generation. When free-swimming F2 fry emerged in an F1 tank, they were transferred to stock tanks; these fry were regarded as 1 month old. The fry were bred in the stock tanks until they reached an age of 6 ± 1 month and were then transferred to F2 tanks at densities of 20–35 fish per tank. The F2 tanks measured 64 × 37 cm with water depth of 13.5 cm. The water temperature was maintained at 26 ºC. As males usually compete for substrates (rocks and shells) to form territories and females also compete for substrates to spawn, no substrates other than heaters and aerators were provided in the F2 tanks to reduce this competition. This also reduced any effects of hiding-space size on body size, if any. The F2 fish were fed three times a day, for 5 days per week. Dead fish and short-body individuals were removed immediately on discovery. The sexes of the F2 fish were determined from the shapes of the genital papillae at the age of 13 months. At this time, the F2 females were euthanized using FA100 anaesthetic (DS Pharma Animal Health, Osaka, Japan) and fixed in > 99 % ethanol. The males were continuously bred in the F2 tanks until they were 16 months old, at which time they were also euthanized and fixed in the same ways. These F2 individuals were sexually mature, i.e., males had white testes and females had non-transparent eggs in the ovaries. We collected 6–16 males and 7–17 females from each of the ten F2 tanks. A total of 206 F2 individuals were obtained, consisting of 100 males and 106 females.

### Body size estimation

The SLs of the fixed F2 fish were measured using CD67-S20PS digital callipers (Mitutoyo, Kanagawa, Japan) under an SMZ 1000 binocular microscope (Nikon, Tokyo, Japan). The SLs of the F2 males ranged from 32.3 to 55.9 mm. The smallest size was within the previously reported size range for adult dwarf males from Wonzye [14]. The largest size almost matched the smallest reported size of adult normal males from Wonzye (56.4 mm SL). The SLs ranged from 24.2 to 40.2 mm in the F2 females. The smallest and largest sizes were within the body size ranges of the adult dwarf and normal females, respectively, from Wonzye [14].

### ddRAD sequencing

The ddRAD libraries were prepared according to a previously described method [43] with some minor modifications. RNA-free total genomic DNA was extracted from the right pectoral fins and body muscles of the F0 male, F0 female, and 206 F2 individuals using a Wizard Genomic DNA Purification Kit (Promega, Madison, WI, USA). The DNA concentration was determined using a Qubit 3.0 Fluorometer (Invitrogen, Carlsbad, CA, USA) and adjusted to 20 ng/µl. Each sample (10 µl) was digested with high-fidelity EcoRI (New England Biolabs, Ipswich, MA, USA; 10 U for each sample) and BglII (Takara Bio, Shiga, Japan; 5 U for each sample) and simultaneously ligated with sequencing adaptors using T4 DNA Ligase (Enzymatics, Beverly, MA, USA) in NEB buffer 2.1. After purification with AMPure XP (Beckman Coulter, CA, USA), each ligated sample was amplified using KAPA HiFi HS ReadyMix (Kapa Biosystems, Wilmington, MA, USA) with primers barcoded with a unique eight-nucleotide sequence. Thermal cycling was initiated at 95 ºC for 3 min, followed by 20 cycles of 98 ºC for 20 s, 65 ºC for 10 s, and 72 ºC for 30 s. The 208 PCR products obtained were pooled in the same volume after purification with AMPure XP. DNA fragments of 320–450 base pairs were retrieved on 2.0 % agarose gel using E-Gel SizeSelect (Life Technologies, Carlsbad, CA, USA). The pooled sample was run in one lane of paired-end 150 + 150-bp sequencing on a HiSeq X sequencer (Illumina, San Diego, CA, USA) at Macrogen (Seoul, South Korea). The sequenced reads were demultiplexed using CASAVA 1.8.2 (Illumina, San Diego, CA, USA).

### Processing of ddRAD sequences

A *de novo* orthology search was conducted using the Stacks ver. 2.54 software package [44, 45]. For each individual, only ‘stacks’ with three or more identical ddRAD sequences were used, and stacks with one or two different sites were identified as orthologous loci. The orthologous loci of the F0 male and F0 female were used to identify SNPs, allowing for the detection of orthologs with one or two different sites between the F0 individuals. The identified SNPs were genotyped in the F2 individuals. Loci were only accepted when allelic stacks were recovered in > 60 % of the samples. In cases of two or more SNPs at a single locus, only the first SNP was used. To avoid ambiguous identification of the origin of the F2 alleles (i.e., from the F0 male or F0 female), SNPs with alleles shared by the F0 male and F0 female were removed using a custom Perl script.

### Linkage map construction

A linkage map was created using Lep-MAP3 [46]. SNPs in which the genotypes extremely departed from the expected segregation pattern (aa:ab:bb = 1:2:1, *p* < 0.0001) were discarded. The remaining SNPs were separated into LGs with a limitation of LOD score = 10 and a recombination rate = 0.05. In each LG, the most likely map with the largest likelihood score was selected from 100 estimations initiated with different random seeds. The linkage map created was visualized using MapChart 2.32 [47].

### QTL mapping

QTL for body size were detected using R/qtl2 [48]. First, pseudomarkers at intervals of 1 cM were created based on the LGs. A genome scan was then conducted for both the original ddRAD SNP markers and the pseudomarkers, taking into consideration the random polygenic effect. This genome scan requires kinship matrices. The kinship matrix of each LG was calculated using pseudomarkers from all the other LGs to eliminate any effect of varying marker density. As the log_10_(SL) significantly differed among tanks as well as between sexes due to a confounding tank effect [two-way analysis of variance (ANOVA): *F*_9,186_ = 5.91, *p* < 0.0001 among tanks, *F*_1,186_ = 533, *p* < 0.0001 between sexes, *F*_9,186_ = 1.08, *p* > 0.05 for the interaction between tank and sex], tank and sex effects were included in the genome scan as additive covariates. Ten thousand permutations were conducted to estimate the significant and suggestive levels of the LOD scores, in which statistical evidence was expected to occur 0.05 and one time, respectively, in a genome scan [23]. A 95 % Bayes credible interval for each QTL was estimated. The body-size difference among genotypes of QTL was visualized using residuals from two-way ANOVA on log_10_(SL) without considering interaction to remove tank and sex effects. The proportion of phenotypic variance explained (PVE) by each QTL was calculated based on the residuals: PVE = (sum of squares between genotypes) / (sum of squares total).

The ddRAD loci were subjected to blast [49] searches using the megablast option against the genome of *O. niloticus* (F11D_XX) in the National Center for Biotechnology Information (NCBI) database. When blast search found more than one significant similar locus to a query ddRAD locus, only the most similar locus that has *E*-value smaller than 1/10^10^ times that of the second likely locus was regarded as the corresponding locus of the query. Otherwise, it was regarded as ‘unidentified’, as well as the cases that no significant similar loci were found and that the most similar locus was on an unplaced genomic scaffold of *O. niloticus*.

## Supplementary Information

**Additional file 1: Supplementary Figure S1. **A linkage map of *Telmatochromis temporalis*. Twenty-two linkage groups (LGs) consist of 708 double-digested restriction-site associated DNA (ddRAD) markers that were generated from a hybrid cross between the *T. temporalis *normal and dwarf morphs. **Supplementary Figure S2. **Comparison of double-digested restriction-site associated DNA (ddRAD) locus positions between *Telmatochromis temporalis *and *Oreochromis niloticus*. Sixteen linkage groups (LGs) that do not contain significant or suggestive quantitative trait locus (QTL) for body size are shown. Question mark indicates markers for which the positions on the *O. niloticus *LG were not identified. See Fig. 3 for the other LGs with QTL. **Supplementary Table S1. **Results of blast search for ddRAD loci of *Telmatochromis temporalis *against an *Oreochromis niloticus *genome.

## Data Availability

The ddRAD sequence data are available at DDBJ (accession no. DRA011699). https://ddbj.nig.ac.jp/DRASearch/submission?acc=DRA011699.

## Abbreviations

ANOVA: Analysis of variance
CI: Credible interval
DDBJ: DNA Data Bank of Japan
ddRAD: Double-digested restriction-site associated DNA
*egr1*: Early growth response 1
*ghr*: Growth hormone receptor
LG: Linkage group
*igf1*: Insulin-like growth factor 1
*igfbp4*: Insulin-like growth factor binding protein 4
LOD: Logarithm of odds
NCBI: National Center for Biotechnology Information
*nr2c2*: Nuclear receptor subfamily 2 group C member 2
PVE: Proportion of phenotypic variance explained
QTL: Quantitative trait loci
SL: Standard length
*smad7*: SMAD family member 7
SNP: Single nucleotide polymorphism
