# Supplementary material for: Mapping of quantitative trait loci underlying a magic trait in ongoing ecological speciation
Source: BMC Genomics. 2021 Aug 12;22:615. doi: 10.1186/s12864-021-07908-4 (PMC8361645; doi:10.1186/s12864-021-07908-4)
Supplement: Supplementary file 1 — Additional file 1: Supplementary Figure S1. A linkage map of Telmatochromis temporalis. Twenty-two linkage groups (LGs) consist of 708 double-digested restriction-site associated DNA (ddRAD) markers that were generated from a hybrid cross between the T. temporalis normal and dwarf morphs. Supplementary Figure S2. Comparison of double-digested restriction-site associated DNA (ddRAD) locus positions between Telmatochromis temporalis and Oreochromis niloticus. Sixteen linkage groups (LGs) that do not contain significant or suggestive quantitative trait locus (QTL) for body size are shown. Question mark indicates markers for which the positions on the O. niloticus LG were not identified. See Fig. 3 for the other LGs with QTL. Supplementary Table S1. Results of blast search for ddRAD loci of Telmatochromis temporalis against an Oreochromis niloticus genome. [file 12864_2021_7908_MOESM1_ESM.pdf]

Mapping of quantitative trait loci underlying a magic trait in ongoing ecological speciation

Tetsumi Takahashi, Atsushi J. Nagano, Teiji Sota

Supplementary Fig. S1

Supplementary Fig. S2

Supplementary Table S1

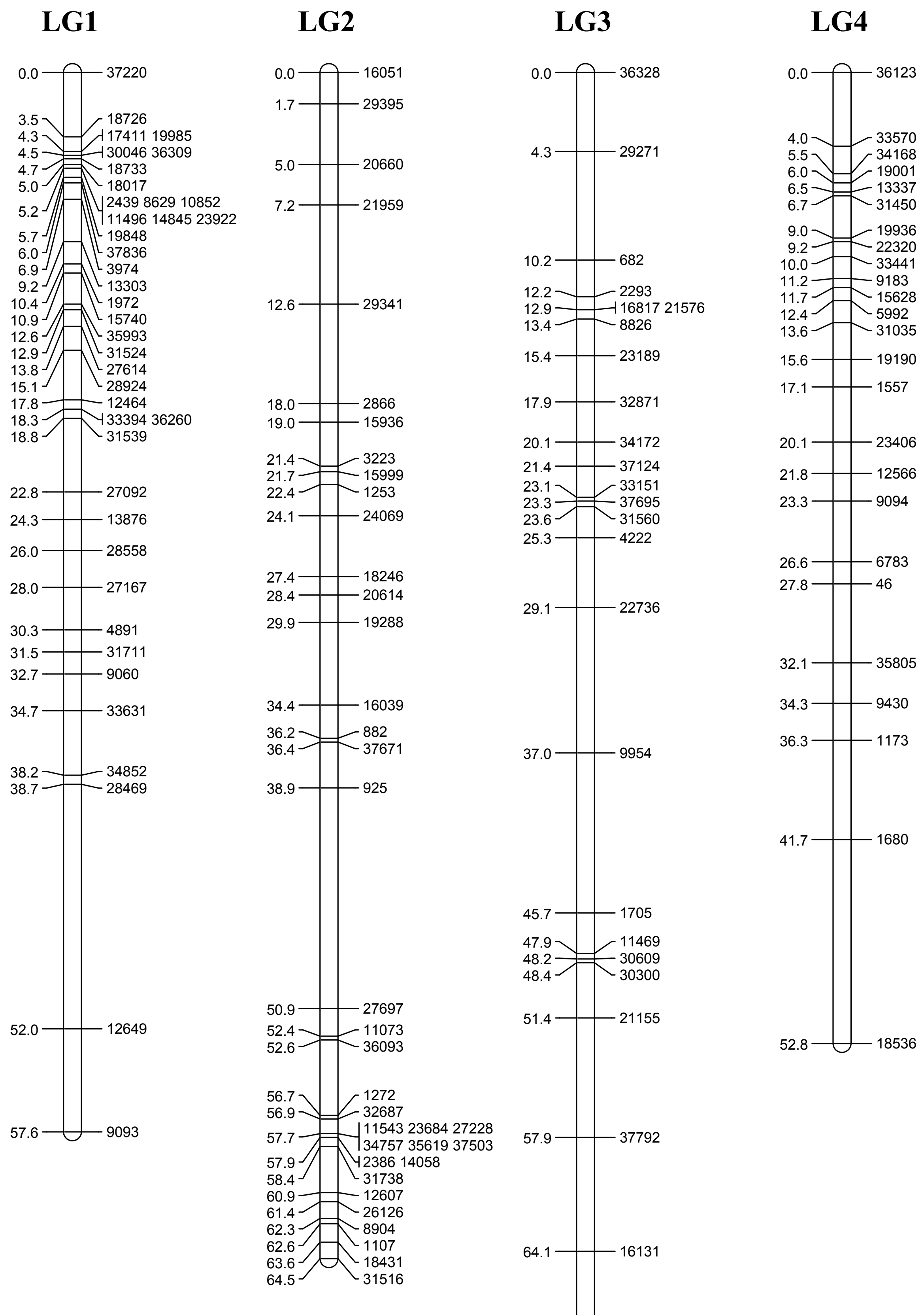
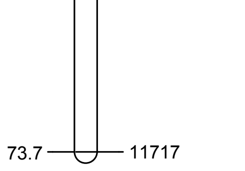

Supplementary Fig. S1 A linkage map of *Telmatochromis temporalis*. Twenty-two linkage groups (LGs) consist of 708 double-digested restriction-site associated DNA (ddRAD) markers that were generated from a hybrid cross between the *T. temporalis* normal and dwarf morphs.

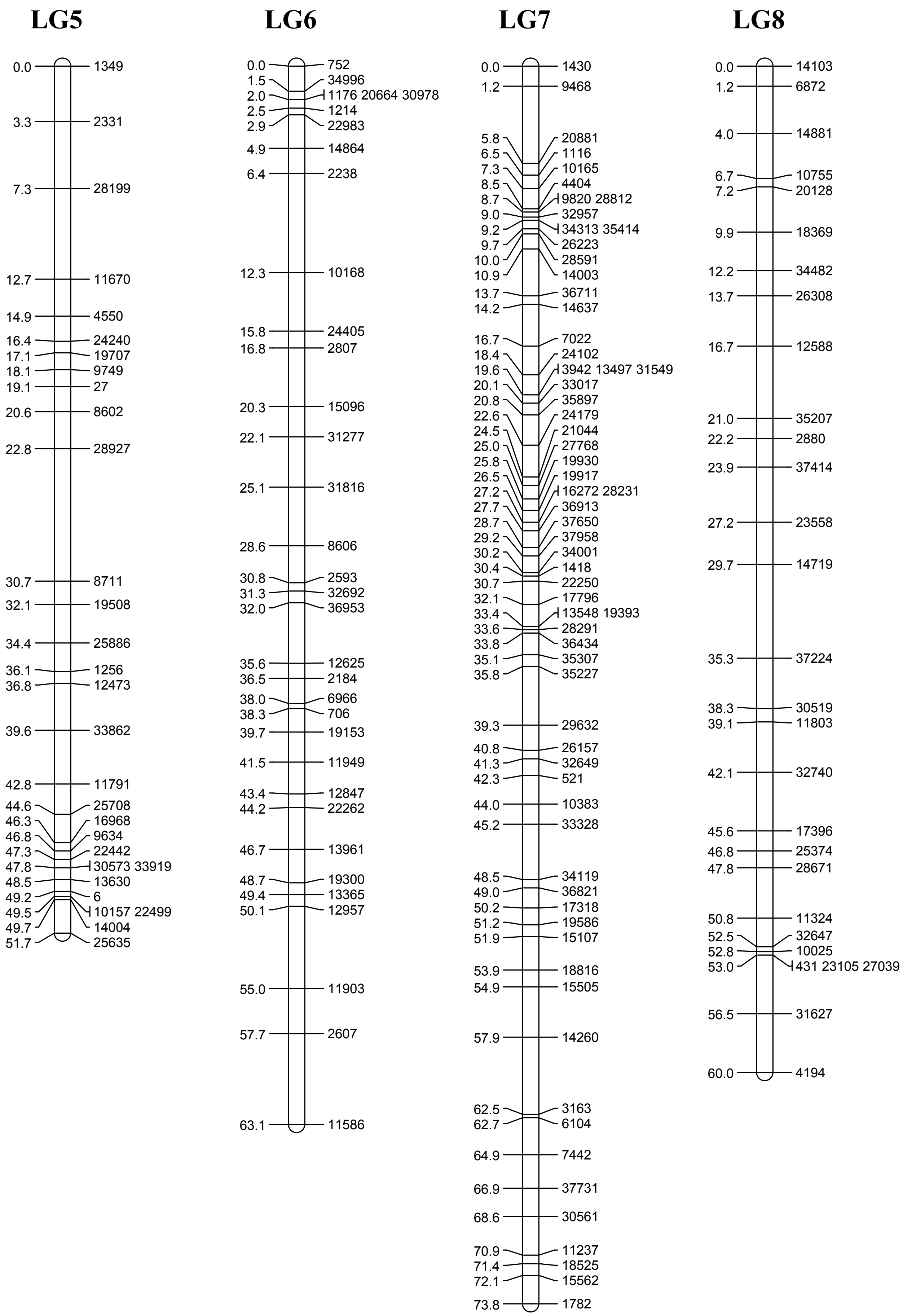

Supplementary Fig. S1 (continued)

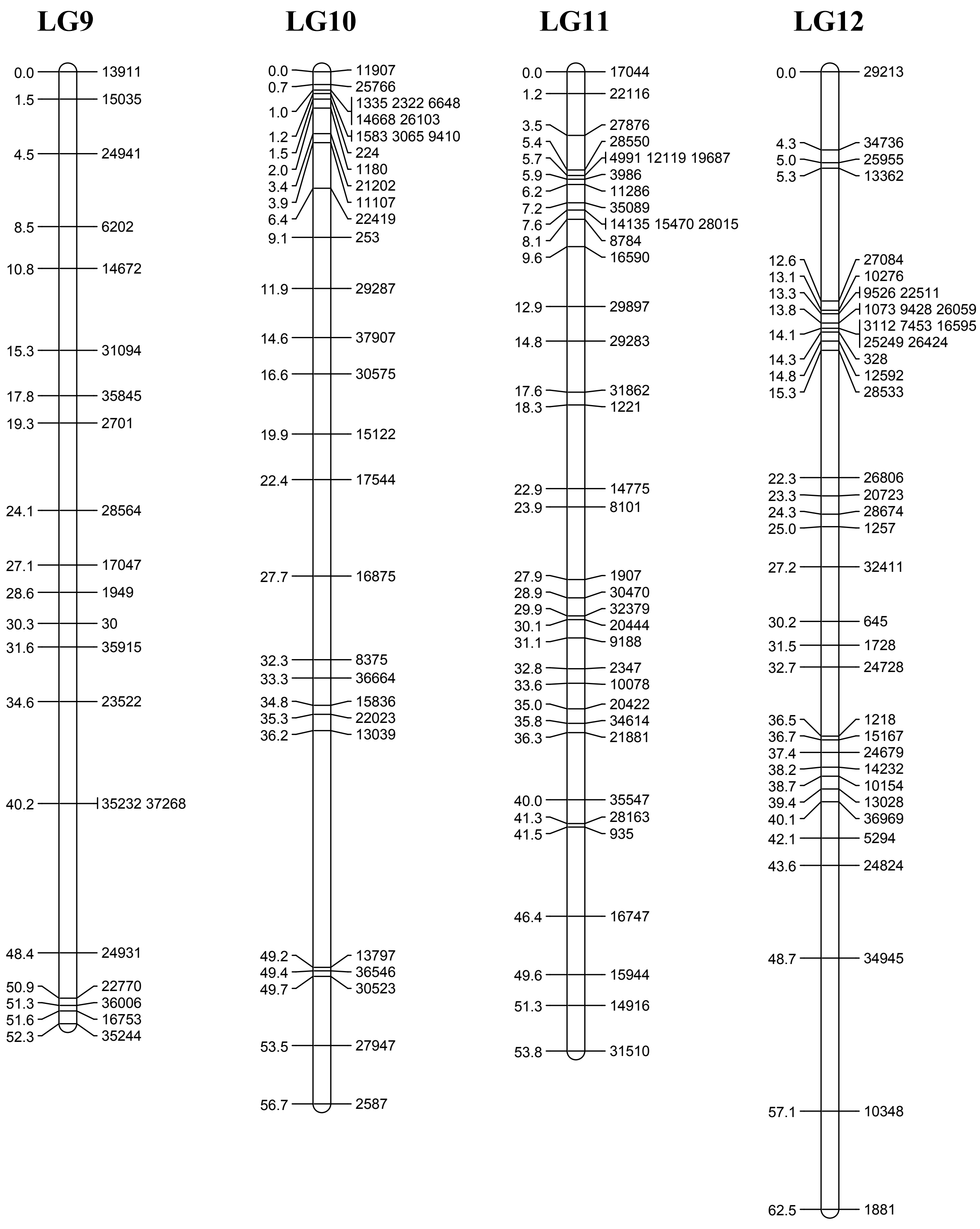

Supplementary Fig. S1 (continued)

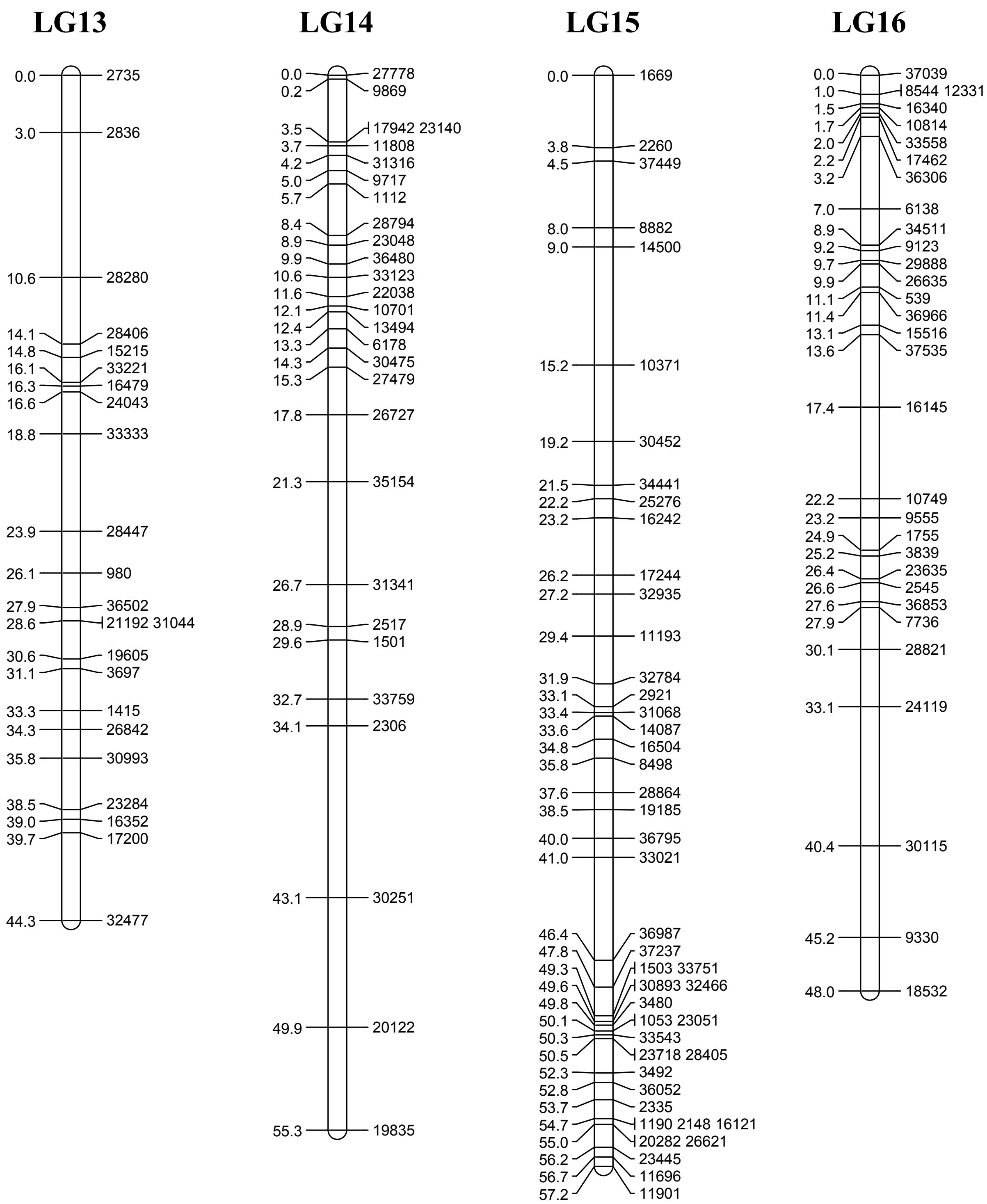

Supplementary Fig. S1 (continued)

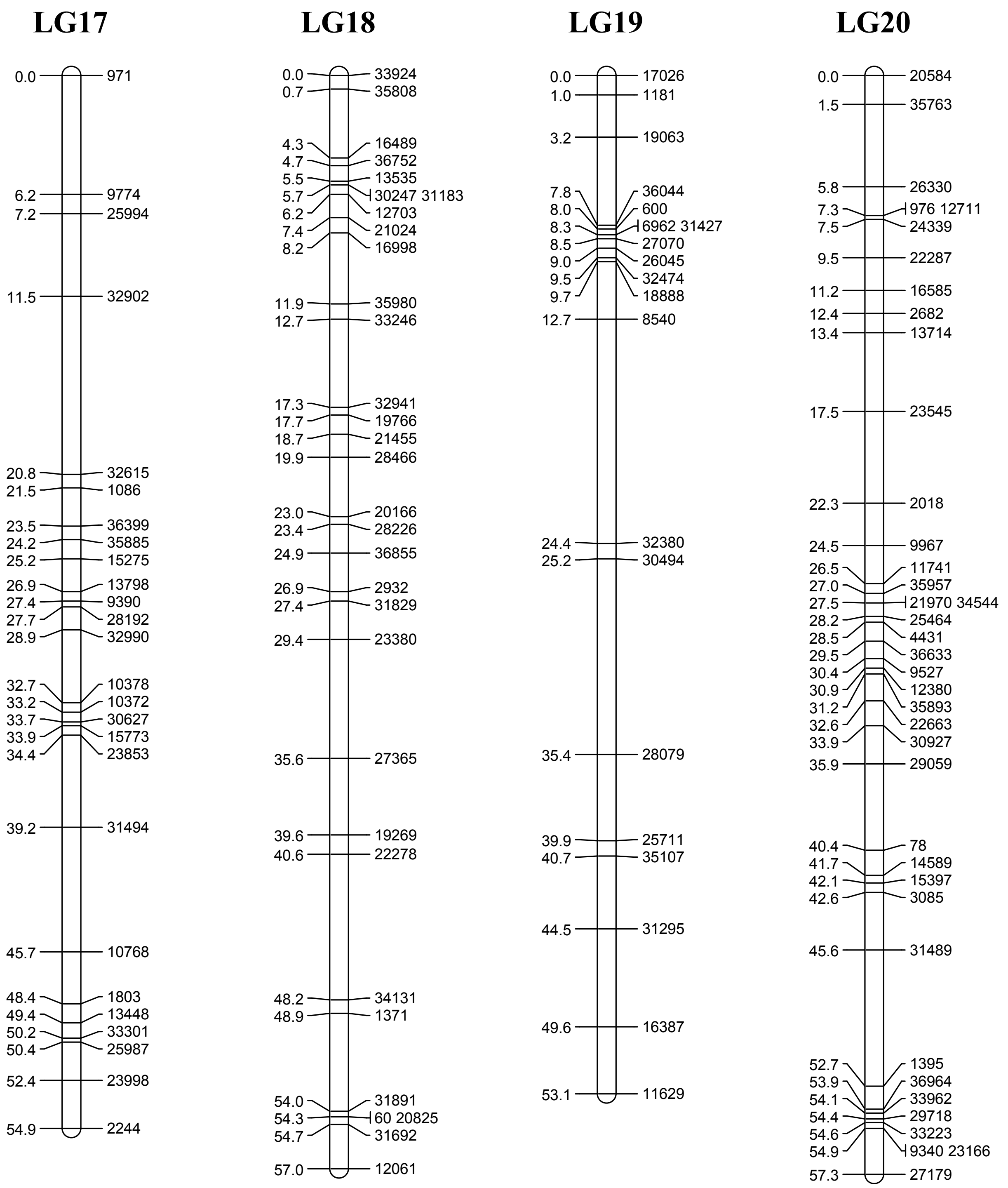

Supplementary Fig. S1 (continued)

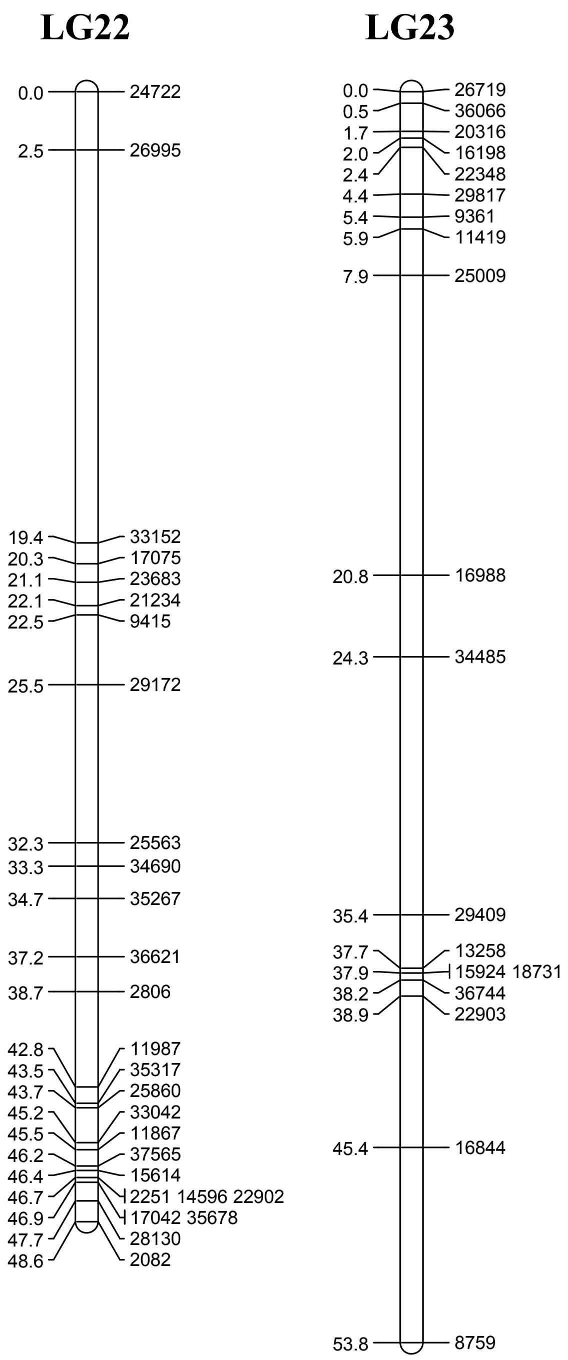

Supplementary Fig. S1 (continued)

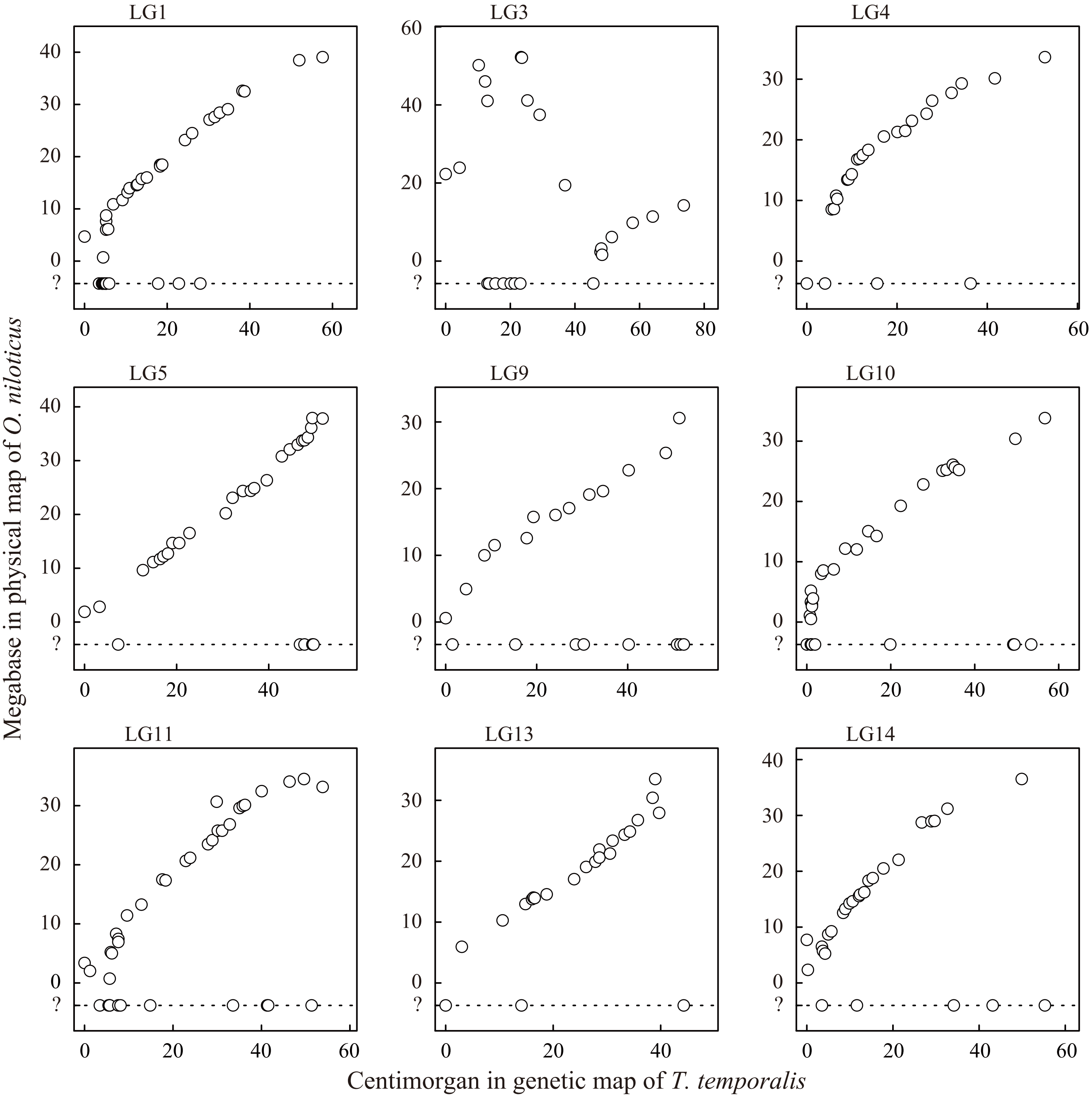

Supplementary Fig. S2 Comparison of double-digested restriction-site associated DNA (ddRAD) locus positions between *Telmatochromis temporalis* and *Oreochromis niloticus*. Sixteen linkage groups (LGs) that do not contain significant or suggestive quantitative trait locus (QTL) for body size are shown. Question mark indicates markers for which the positions on the *O. niloticus* LG were not identified. See Fig. 3 for the other LGs with QTL.

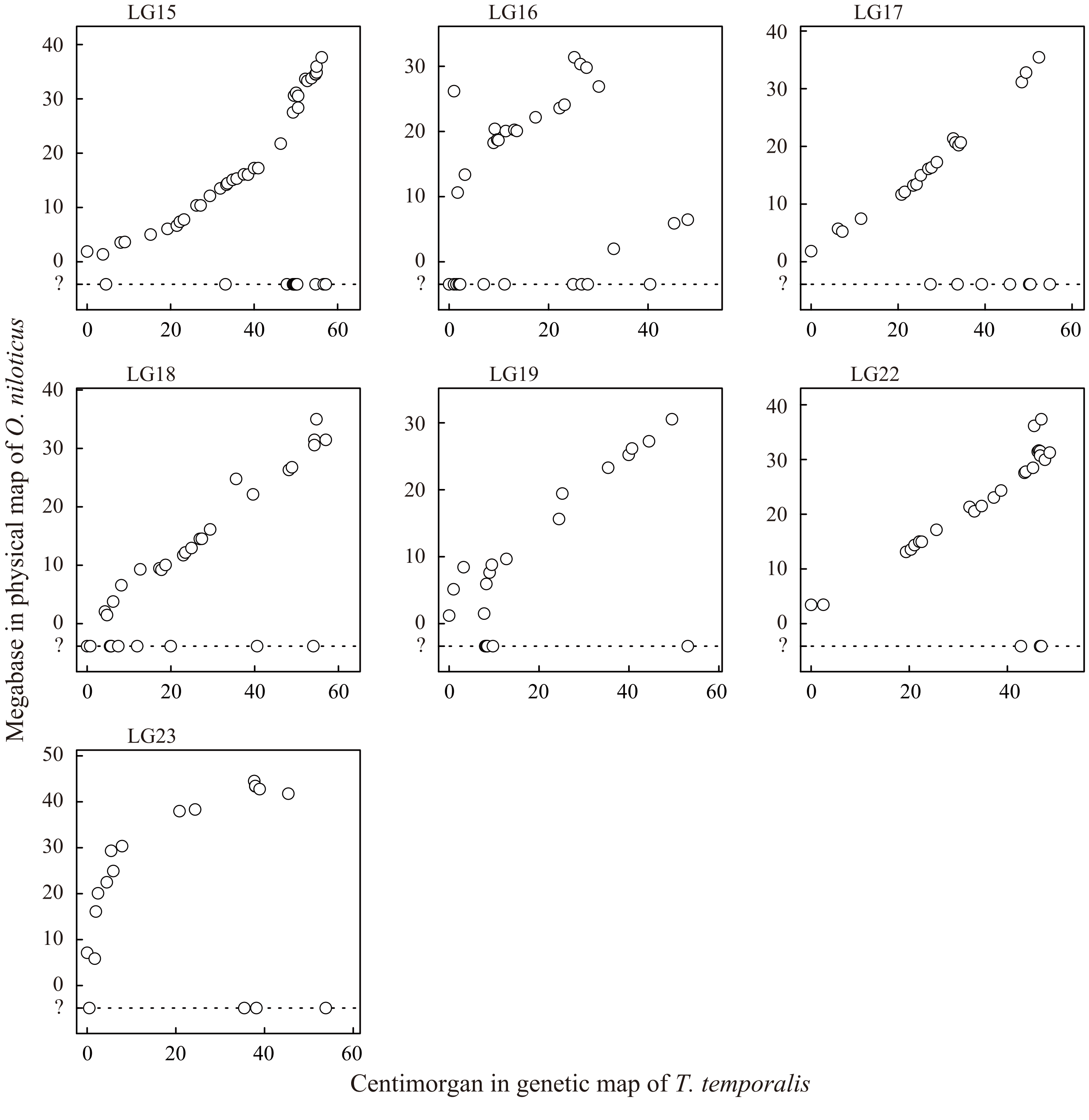

Supplementary Fig. S2 (continued)

Supplementary Table S1 Results of blast search for ddRAD loci of *Telmatochromis temporalis* against an *Oreochromis niloticus* genome.

LGs of *T. temporalis*	LGs of *O. niloticus*																						Total
	1	2	3	4	5	6	7	8	9	10	11	12	13	14	15	16	17	18	19	20	22	23	?	
1	27	0	0	0	0	0	0	0	0	0	0	0	0	0	0	0	0	0	0	0	0	0	13	40
2	0	34	0	0	0	0	0	0	0	0	0	0	0	0	0	0	0	0	0	0	0	0	4	38
3	0	0	17	0	0	0	0	0	0	0	0	0	0	0	0	0	0	0	0	0	0	0	8	25
4	0	0	0	21	0	0	0	0	0	0	0	0	0	0	0	0	0	0	0	0	0	0	4	25
5	0	0	0	0	25	0	0	0	0	0	0	0	0	0	0	0	0	0	0	0	0	0	5	30
6	0	0	0	0	0	21	0	0	0	0	0	0	0	0	0	0	0	0	0	0	0	0	13	34
7	0	0	0	0	0	0	57	0	0	0	0	0	0	0	0	0	0	0	0	0	0	0	9	66
8	0	0	0	0	0	0	0	24	0	0	0	0	0	0	0	0	0	0	0	0	0	0	5	29
9	0	0	0	0	0	0	0	0	13	0	0	0	0	0	0	0	0	0	0	0	0	0	8	21
10	0	0	0	0	0	0	0	0	0	23	0	0	0	0	0	0	0	0	0	0	0	0	9	32
11	0	0	0	0	0	0	0	0	0	0	27	0	0	0	0	0	0	0	0	1	0	0	10	38
12	0	0	0	0	0	0	0	0	0	0	0	31	0	0	0	0	0	0	0	0	0	0	8	39
13	0	0	0	0	0	0	0	0	0	0	0	0	20	0	0	0	0	0	0	0	0	0	3	23
14	0	0	0	0	0	0	0	0	0	0	1	0	0	23	0	0	0	0	0	0	0	0	4	28
15	0	0	0	0	0	0	0	0	0	0	0	0	0	0	35	0	0	0	0	0	0	0	11	46
16	0	0	0	0	0	0	0	0	0	0	0	0	0	0	0	20	0	0	0	0	0	0	11	31
17	0	0	0	0	0	0	0	0	0	0	0	0	0	0	0	0	19	0	0	0	0	0	7	26
18	0	0	0	0	0	0	1	0	0	0	0	0	0	0	0	0	0	22	0	0	0	0	9	32
19	0	0	0	0	1	0	0	0	0	0	0	0	0	0	0	0	0	0	15	0	0	0	4	20
20	0	0	0	0	0	1	0	0	0	0	0	0	0	0	0	0	0	0	0	33	0	0	5	39
22	0	0	0	0	0	0	0	0	0	0	0	0	0	0	0	0	0	0	0	0	24	0	3	27
23	0	0	0	0	0	0	0	0	0	0	0	0	0	0	0	0	0	0	0	0	0	15	4	19

ddRAD: Double-digested restriction-site associated DNA; LGs, linkage groups; ?, unidentified loci, including loci to which no significantly similar *O. niloticus* scaffolds were found, loci identified to unplaced genomic scaffolds of *O. niloticus*, and loci to which the E-value of the most likely significantly similar scaffolds was larger than $1/10^{10}$ times of that of the second one.
